# Assessment of Intestinal Parasites, Enteric Bacterial Infections, and Antimicrobial Susceptibility among Street Food Handlers in Jimma Town, Southwest Ethiopia

**DOI:** 10.1155/2022/5483367

**Published:** 2022-04-11

**Authors:** Tibeso Gemechu, Teferi Eshetu, Tesfaye Kassa, Habtemu Jarso

**Affiliations:** ^1^Department of Medical Laboratory Science, College of Health Sciences, Bule Hora University, Bule Hora, Oromia, Ethiopia; ^2^Departments of Medical Laboratory Science, Faculty of Health Sciences, Institute of Health, Jimma University, Jimma, Ethiopia; ^3^Department of Public Health, School of Health Science, Madda Walabu University Shashemene Campus, Shashemene, Ethiopia

## Abstract

**Background:**

Food-borne infections are common public health problems worldwide. A street food handler with poor personal hygiene contributes to the transmission of intestinal parasites and enteric bacteria to the public via contaminated foods. In Ethiopia, health risks associated with street food are common. Previous studies in this area are scanty. Hence, the aim of this study was to determine the prevalence of intestinal parasites, enteric bacterial infections, and antimicrobial susceptibility among street food handlers in Jimma town.

**Methods:**

A cross-sectional study was conducted from October to December 2020 among 260 street food handlers in Jimma town. A semi-structured questionnaire was used to collect data through face-to-face interviews. About 3 grams of the fecal specimen were collected from each food handler for bacterial culture and concentration techniques. The data were entered into Epi-Data 3.1 and analyzed by SPSS version 20. Associated factors were identified by using binary logistic regression analysis. A statistically significant association was determined at a *p*-value less than 0.05.

**Results:**

The overall prevalence of intestinal parasites and enteric bacterial pathogens was 39.2% (33.3%–45.2%) and 8.85% (5.4%–12.3%), respectively. *Ascaris lumbricoides* (18.5%) and *Salmonella* (8.1%) were the most predominant parasite and enteric bacterial isolates, respectively. Not trimming fingernails (AOR = 2.884; 95% CI: 1.682–4.945) and not washing hands with soap after toilet (AOR = 3.342; 95% CI: 1.939–5.761) were factors associated with increased risk of infection by intestinal parasites or enteric bacterial pathogens. All *Salmonella* and *Shigella* isolates were 100% resistant to ampicillin.

**Conclusion:**

The infection with intestinal parasites and enteric bacterial pathogens detected in this study indicated that street food handlers may serve as sources of pathogens/parasites for transmission and experience morbidities due to the infections. Therefore, periodic medical checkups and creating awareness of personal hygiene are mandatory to reduce the risk of infections.

## 1. Introduction

Food-borne pathogens refer to a group of infectious microorganisms including parasites that cause disease in humans via food or food-borne routes. Contamination of processed food during serving of food to the customers frequently occurred by street food handlers with poor personal hygiene. This contributes to food-borne illnesses and outbreaks [1]. An estimated 2.5 billion people around the world eat street food every day. As a result, food-borne diseases caused by pathogenic bacteria and parasites are becoming a major health problem associated with street foods [2, 3]. Bacteria, viruses, and parasites are responsible for food-borne diseases. Salmonellosis, shigellosis, diarrhea associated with *Escherichia coli*, and staphylococcal food poisoning are the leading causes of morbidity and mortality from bacterial pathogens [4].

The World Health Organization (WHO) estimated that 31 food-borne pathogens caused 600 million illnesses and 420,000 deaths in 2015. In this estimate, nontyphoidal *Salmonella enterica* resulted in 230,000 deaths followed by *Salmonella typhi* which caused 52,472 deaths. Intestinal parasites responsible for food-borne diseases include *Giardia lamblia*, *Entamoeba histolytica*, Cryptosporidium species, and various helminthes. In 2015, the WHO estimated that 13 million food-borne illnesses are caused by helminthes leading to 45 thousand deaths. On the other hand, intestinal protozoa such as *Entamoeba histolytica* and *Giardia lamblia* were estimated to cause disease in about 28,000,000 people [5]. In Africa particularly, the food-borne illness continues to be a major health threat, especially for vulnerable groups, such as infants, pregnant women, and immune-compromised individuals [6]. In these countries, bacteria and parasites constitute the major causes of food-borne diseases which are frequently transmitted through food, nails, and fingers contaminated with feces [7].

Street food handlers play a key role in the spread of the enteric pathogen to the customers due to their poor personal hygiene and sanitation. Besides this, they prepare and sell food in an overcrowded area where a higher probability of food contamination is expected. A study showed that street food handlers with poor personal hygiene and poor sanitary conditions can be infected by different enteric pathogens which may have the probability to be transmitted to their customers [8]. Few studies have been conducted on the prevalence of either intestinal parasites or enteric bacteria among street food handlers worldwide. On these notes, the prevalence of intestinal parasites was 21.6% in Ghana [9] and 56.7% in Cameroon [8]. One published study from Ethiopia reported that the prevalence of *Salmonella* and *Shigella* among street food handlers was 8.7% [10].

There is limited data on the prevalence of enteric pathogenic bacteria and parasites among street food handlers in Africa including Ethiopia. Hence, the aim of this study was to assess the prevalence of intestinal parasitic and enteric bacterial infections among street food-handlers in Jimma town. The results of this study will be used as part of the evidence to come up with a better solution to the problem.

## 2. Materials and Methods

### 2.1. Study Area and Study Period

The study was conducted among street food handlers from October to December 2020 in Jimma town. The town is located 356 km from Addis Ababa in the southwestern part of Ethiopia. The total population of Jimma town was estimated to be 205,384 in 2018. Of these 104,745 were females. In the town, there were four health centers and two hospitals. Street food handlers were involved in vending sambusa, cooked potato, injera, bread, bombolino, and other foods in the town. But their exact number is not known because of the absence of legally registered data at the town level.

### 2.2. Study Design

A community-based cross-sectional study was carried out to assess the prevalence of enteric pathogenic bacteria and intestinal parasite infection among street food handlers in Jimma town.

### 2.3. Source Population

All adult street food handlers working in Jimma town at the time of data collection.

### 2.4. Study Population

All adult street food handlers in Jimma town who were available during the data collection and fulfilled inclusion criteria.

### 2.5. Inclusion and Exclusion Criteria

All adult (age ≥18 years) street food handlers who were willing to participate in the study. Street food handlers who have taken antihelminthic, antiprotozoal, and antimicrobial drugs two weeks prior to sample collection were excluded [11].

### 2.6. Sample Size

The sample was calculated using the single population proportion formula with an assumption of 95% confidence level, 5% margin of error, and a population proportion of 21.6% taken from Ghana for parasites [9] and 8.7% taken from Dire Dawa city for enteric bacteria [10]. A larger sample size (260) was taken from the two calculated sample sizes. Considering a 10% non-response rate, the sample size was increased to 286.

### 2.7. Sampling Technique

Since street food vendors were not legally registered and did not have a permanent work site, it was difficult to know their exact total number. Therefore, a convenient sampling technique was used. All available street food handlers in the town were included consecutively in the study until the sample size was fulfilled.

### 2.8. Data Collection and Laboratory Processing

The socio-demographic data were collected from participants through face-to-face interviews using a semi-structured questionnaire. The stool was collected in clean, dry, and leak-proof stool cups from each selected street food handler. The stool sample was transported for examination immediately after collection. For enteric bacteria, approximately 1 g of stool sample was immediately transferred into the Cary Blair transporting medium, labeled, and transported within one hour of collection in an ice-packed cold box (4°C) to the examination area [13].

### 2.9. Microscopic Examination of Stool

The stool was observed macroscopically (physically) for the presence of adult stages of some intestinal helminthes, consistency, and color. After visual assessment of the stool, the intestinal parasite was examined microscopically from each stool specimen using both direct smears mounted in saline and the formal ether concentration procedure as recommended [13].

### 2.10. Culture and Identification of Bacteria

A loop full fecal suspension was added to selenite F broth and incubated for 18 hours. After incubation, a loop full of the suspended colonies was inoculated into xylose lysine deoxycholate agar (High Media, India). Then, it was incubated aerobically for 24 hours at 37°C. After overnight incubation, the growth of *Salmonella* and *Shigella* species was detected by their characteristic appearance on XLD agar (*Salmonella*: red with or without a black center; *Shigella*: red/pink colonies) [14]. Five series of biochemical tests such as Klinger iron agar (KIA), Simmons citrate agar, sulfide indole motility test (motility, H2S production, indole), lysine iron agar (LIA), and urease test were used for final identification of bacterial isolate [14].

### 2.11. Antimicrobial Susceptibility Test

Antimicrobial susceptibility testing was performed using the Kirby-Bauer disk diffusion method as described by the Clinical Laboratory Standards Institute [15]. In brief, the pure culture was transferred to a tube containing 5 ml of sterile normal saline (0.85% NaCl) and mixed gently until it formed a homogeneous suspension. The turbidity of the suspension was adjusted to an optical density equivalent to 0.5 McFarland standards. A sterile cotton swab was then dipped into the suspension and the excess was removed by gentle rotation of the swab against the surface of the tube. The swab was distributed evenly over the entire surface of the Mueller–Hinton agar (Oxoid, UK). The inoculated plates were left at room temperature to dry for 3 to 5 minutes. Six antimicrobial discs (Oxoid, UK) such as ampicillin (10 *μ*g), trimethoprim-sulfamethoxazole (23.75/1.25 *μ*g), ciprofloxacin (5 *μ*g), gentamicin (10 *μ*g), ceftriaxone (30 *μ*g), and Ddxycycllin (30 *μ*g) were placed aseptically on the inoculated plate using sterile forceps. After 24 hours of incubation at 37°C, the zone of inhibition including disk was measured by using a digital caliper to the nearest whole millimeters and interpreted as sensitive, intermediate, or resistant based on interpretive breakpoints [15].

### 2.12. Quality Assurance

A questionnaire was developed initially in English and translated into local languages (*Amharic and Afaan Oromo*) by consulting language experts. The data were collected by the principal investigator and assistant trained data collector. The completeness of each of the questionnaires was checked before data analysis. The specimen was checked for a serial number, quality, and procedures of collection. The obtained sample was taken to the laboratory immediately after collection. Two experienced laboratory professionals were involved in the culture and microscopic examinations of stool. The sterility of each medium was checked by incubating them overnight at 37°C.

The media which showed growth was discarded and replaced by new media that did not show any growth following overnight incubation. The reference of American type culture collection strains such as E. *coli* (ATCC 25922) and P. *aeruginosa* (ATCC 27853) was used as quality control throughout the study for culture and antimicrobial susceptibility tests.

### 2.13. Data Analysis

The data were entered into Epi-Data version 3.1 and exported to SPSS version 20 for analysis. Bivariate and multivariable logistic regressions were used to assess the association between dependent variables and independent variables. The odds ratio was calculated to measure the strength of association. A variable with a *p*value < 0.25 in the bivariate logistic regression analysis was further analyzed using the multivariable logistic regression. A *p*-value less than 0.05 in the multivariable logistic regression analysis was considered statistically significant.

### 2.14. Ethical Consideration

Ethical clearance and permission were obtained from the institutional ethical review committee of the Institute of Health, Jimma University, before starting data collection. A legal support letter from Jimma University was given to Jimma town Health Bureau and a permission letter was obtained. The objective of the study was clarified to respondents, and written consent was obtained from each participant. The information obtained was kept confidential, and study participants were informed about the importance of the study. Those participants found to be infected were linked to the nearby health facility to get appropriate treatment.

## 3. Results

### 3.1. Socio-demographic Characteristics of Street Food Handlers

A total of 260 food handlers who fulfilled inclusion criteria were enrolled in this study. Most (237, 91.2%) of the study participants were females. The age of the participants ranged from 18 to 56 years with a mean age of 26.93 years. The majority (141, 54.2%) of the study participants were within the age range of 21–30 years old. The majority (142, 54.6%) of the study participants are currently married. The majority (152, 58.5%) of the study participants have served as street food handlers for 1–5 years. Nearly half (129, 49.6%) of the street food handlers were able to read and write. Almost half (131, 50.4%) of the street food handlers had an average monthly income of 500 up to 999 Ethiopian Birr. About 98 (37.7%) street food handlers earn an average monthly income of less than 500. No street food handler has visited a health facility for a medical checkup (Table 1).

### 3.2. Prevalence and Associated Factors of Intestinal Parasites and Enteric Bacterial Infections

**Figure 1:**
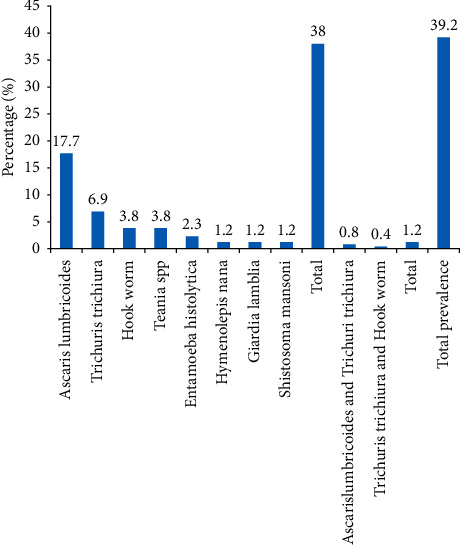
Distribution of intestinal parasites among street food handlers in Jimma town, Southwest Ethiopia (*n* = 260), Oct–Dec 2020.

Out of 260 stool samples examined, 102 stool specimens were positive for different intestinal parasites making the overall prevalence 39.2% (95% CI: 33.3%–45.2%). Of the positive individuals, 99 (38%) had single infections and 3 (1.2%) had mixed infections. Eight different intestinal parasites were identified. The predominant intestinal parasitic infections were *Ascaris lumbricoides* (46, 17.7%) followed by *Trichuris trichiura* (18, 6.9%), *Hookworm* (10, 3.8%), Taenia spp. (10, 3.8%), *Entamoeba histolytica* (6, 2.3%), *Giardia lamblia* (3, 1.2%), *Hymenolepis nana* (3, 1.2%), and *Schistosoma mansoni* (3, 1.2%). Of mixed infections, *Ascaris lumbricoides* and *Trichuri trichiura* (2, 0.8%) were predominant followed by *Trichuris trichiura* and hookworm (1, 0.4%) (Figure 1). From 260 stool samples cultured for *Salmonella* and *Shigella*, 23 stool specimens were positive making the overall prevalence of 8.85% (95% CI: 5.39%–12.3%). *Salmonella* was isolated from 21 (8.08%) and *Shigella* was isolated only from 2 (0.77%) of the study participants.

Five variables were candidates for multiple logistic regression with a *p*-value of less than 0.25. Finally, two variables showed significant association with a *p*-value less than 0.05 on the multiple logistic regression model. Variables significantly associated with infection of intestinal parasites or enteric bacteria were fingernail trimming and washing hands with soap after toilet (*p* < 0.001). According to these findings, a street food handler with untrimmed fingernails was approximately three times more likely (AOR = 2.884, 95% CI: 1.682–4.945) to be infected with intestinal parasites or enteric bacteria compared to a street food handler with trimmed fingernails during the data collection. A street food handler who did not wash his/her hands after the toilet was three times more likely (AOR = 3.342, 95% CI: 1.939–5.761) to acquire intestinal parasite or enteric bacterial infections compared to a street food handler who washed his/her hands with soap after the toilet (Table 2).

### 3.3. Antimicrobial Susceptibility Patterns of *Salmonella* and *Shigella* Isolates

In this study, 21 *Salmonella* and 2 *Shigella* were isolated and tested against six antimicrobial agents. The *Salmonella* isolates were 100% (21/21) resistant to ampicillin, 4.8% (1/21) resistant to trimethoprim-sulfamethoxazole and ciprofloxacin, and 9.5% (2/21) resistant to gentamicin. Complete (21/21) sensitivity of *Salmonella* was found for ceftriaxone. Two of the *Shigella* isolates were resistant to ampicillin. On the other hand, two of the isolates were sensitive to ciprofloxacin, doxycycline, ceftriaxone, and trimethoprim-sulfamethoxazole. Four isolates of *Salmonella* (21.7%) showed resistance to more than one antimicrobial agent. Resistance to two different antimicrobial agents was frequently observed on *Salmonella* isolates (4, 17.4%), while *Shigella* isolates did not show resistance to more than one drug (Table 3).

## 4. Discussion

Street food handlers might be carriers of food-borne enteric bacteria and intestinal parasites infections which play a key role to spread to the public consuming that food. The spread of food-borne infections by food handlers is a common problem globally [16]. We conducted a study to determine the prevalence of intestinal parasites and bacterial infections among street food handlers. The prevalence of intestinal parasites was 39.2%. The current finding was in agreement with studies conducted in Yebu town (44.1%) [17], Bahirdar town (41.1%) [18], Southern Ethiopia (36%) [19], and Addis Ababa University (45.3%) [20]. Low prevalence of intestinal parasites was reported from Ghana (21.5%) [9], Aksum town (14.5%) [21], Chagni town (14.8%) [22], Motta town (26.7%) [23], Sodo University (23.6%) [24], and Sodo town (33.68%) [25] compared to current study. Moreover, a high prevalence of intestinal parasites was reported from Cameroon (56.7%) [8], Mekele University (52.4%) [26], and Neqemte town (52.1%) [27] compared to the current finding.

In this study, the dominant parasite identified was *Ascaris lumbricoides* (18.5%) followed by *Trichuris trichiura* (8.1%). This was similar to the study in Yebu town which reported *Ascaris lumbricoides* as a predominant parasite (17.8%) [17]. A lower prevalence of *Ascaris lumbricoides* was reported from Bahirdar town (11.7%) [18] and Southern Ethiopia (9.27%) [19] compared to the current study. The prevalence of *Trichuris trichiura* (6.9%), the second predominant parasite in the current study, was in agreement with a study in Yebu town (5.9%) [17]. The prevalence of mixed parasite infections in this finding was 1.2% which is comparable with a study from Mekele University which reported 1.3% of mixed intestinal parasite infections [26]. The discrepancy in the prevalence of intestinal parasites in different areas might be due to differences in personal hygiene practices, regular medical checkups, socio-demographic characteristics, and geographical distribution.

The overall prevalence of enteric bacterial pathogen among street food handlers in the current study was 8.85% which agrees with the study in Dire Dawa city (8.7%) [10]. This finding was higher compared to studies in other areas like Debre Markos (5.9%) [28] and Haramaya University (5.04%) [29]. A higher prevalence of *Salmonella* and *Shigella* was reported from a study in Abuja, Nigeria (57.8%) [7] and Gondar town (11.3%) [30] as compared to the current study. The prevalence of *salmonella* was (8.1%) in the current study which was comparable with a study from Dire Dawa city (6%) [10] and Arbaminch (6.9%) [31]. A lower prevalence of *Salmonella* was reported from Motta town (2.5%) [23], Sudan (3.8%) [32], Nigeria (5.5%) [33], Debre Markos (3.6%) [28], and Addis Ababa (3.8%) [34] compared to the current study. However, a higher prevalence of *Salmonella* was reported from Wolyta Sodo (9.1%) [35] compared to the current finding. These variations of *Salmonella* prevalence may be due to personal hygiene and geographical distribution differences.

In this finding, about 0.8% of study participants were carriers of the *Shigella* isolate. This finding agrees with findings from Dire Dawa city (1.7%) [10], Motta Town (1.6%) [23], and Bahirdar University (1.2%) [36]. A higher prevalence of *Shigella* was reported from Gondar University (2.7%) [37] and Gondar town (10.1%) [30] compared to the current study. The prevalence of *Shigella* isolates in the current study was relatively low. However, a study in Addis Ababa University reported no *Shigella* isolate [20]. This discrepancy may be due to the culture media used and geographical distribution.

Of independent factors assessed, trimming fingernails and washing hands with soap after the toilet were significantly associated with intestinal parasites or enteric bacterial infections. Trimming fingernails was a factor independently associated with infection of intestinal parasites or enteric bacteria which was in agreement with studies in Dire Dawa city [10], Yebu town [17], Southern Ethiopia [19], Chagni town [22], Motta town [23], Sodo University [24], and Neqemte town [27]. Food handlers with untrimmed fingernails might contaminate food during serving to the customer if they are infected and can be indicated as potential risks for the public. Another factor significantly associated with the presence of intestinal parasites was washing hands with soap after the toilet which was similar to findings from studies in Dire Dawa city [10], Neqemte town [27], and Arbaminch University [31].

Concerning the antimicrobial resistance profile of the isolates, *Salmonella* showed 100% resistance to ampicillin. This finding was consistent with studies in Bahirdar [18], Motta [23], and Addis Ababa [34]. The sensitivity of the isolate was observed for doxycycline (100%), ciprofloxacin (95%), ceftriaxone (100%), Gentamicin (90.5%), and trimethoprim-sulphamethoxazole (81%). This finding was in line with a study in Dire Dawa city which reported high sensitivity of isolates to ceftriaxone (100%), ciprofloxacin (89.5%), and gentamicin (84.2%) [10]. A study in Addis Ababa University showed high (100%) sensitivity of *Salmonella* to ciprofloxacin and gentamicin which agrees with the current study [20]. The sensitivity of trimethoprim-sulphamethoxazole (81%) in this finding was in line with studies from Motta town (83.3%) [23] and Addis Ababa (85.7%) [34]. However, the lower sensitivity of trimethoprim-sulphamethoxazole (62.5%) was reported from Debremarkos compared to the current finding [28].

In the current study, the *Shigella* isolate was 100% resistant to ampicillin which was consistent with studies from Motta town [23] and Gondar town [30]. This may be due to repeated use of the antimicrobial by the public. For example, ampicillin and other antimicrobials are freely available in local pharmacies, and people could purchase without a prescription and use them with fewer adherences in Ethiopia. On the other hand, the isolate was 100% sensitive to doxycycline, ciprofloxacin, ceftriaxone, gentamicin, and trimethoprim-sulphamethoxazole. This finding agrees with findings from Gondar town [30], Motta town [23], Arbaminch University [31], and Addis Ababa [34]. Hence, these drugs should be prescribed for the patients for the treatment of *salmonella* and *Shigella*.

This study has a few limitations which involve cross-sectional study design and the convenient sampling technique used. A cross-sectional study deals with only point prevalence thus it could not address period variation. The convenient sampling technique employed in this study does not represent the source population. Shortness of the reagents such as deoxycholate agar and Iodine which increase sensitivity for enteric bacteria and protozoa was another limitation for this study.

## 5. Conclusions

The overall prevalence of intestinal parasites or enteric bacterial infections among street food handlers was high in the study area. Trimming fingernails and washing hands with soap after the toilet were factors significantly associated with the presence of intestinal parasites or enteric bacterial pathogen indicating poor personal hygiene of street food handlers. Therefore, periodic medical checkups and health education to street food handlers on personal hygiene should be given by the health bureau to reduce the risk of infections. Both *Salmonella* and *Shigella* isolates showed 100% resistance to ampicillin. In contrast, high sensitivity was observed for other antimicrobials such as doxycycline, ciprofloxacin, ceftriaxone, gentamicin, and trimethoprim-sulphamethoxazole by both isolates.

## Figures and Tables

**Table 1:** Socio-demographic characteristics of street food handlers in Jimma town, Southwest Ethiopia (*n* = 260), Oct–Dec 2020.

Characteristics		Frequency	Percentage (%)
Sex	Male	23	8.8
	Female	237	91.2

Age in years	≤20	61	23.5
	21–30	141	54.2
	31–40	44	16.5
	41–50	13	5
	≥51	1	0.4

Marital status	Single	103	39.6
	Married	141	54.2
	Divorced	10	3.8
	Widowed	6	2.3

Educational status	Cannot read and write	25	9.6
	Read and write only	129	49.6
	Primary education	65	25.0
	High school	38	14.6
	College and above	3	1.2

Income (in birr)	≤500	98	37.7
	501–999	131	50.4
	≥1000	31	11.9

Service years	<1	73	28.1
	1–5	152	58.5
	>5	35	13.5

**Table 2:** Binary logistic regression for factors associated with intestinal parasites or enteric bacterial infections among street food handlers in Jimma town, Southwest Ethiopia (*n* = 260), Oct–Dec 2020.

Independent variables		Presence of intestinal parasite or bacteria		COR (95%CI)	AOR (95%CI)
		Positive no (%)	Negative no (%)		
Eating raw vegetable or fruit	No	43 (39.4%)	66 (60.6%)	1	
	Yes	71 (47%)	80 (53%)	1.362 (0.826,2.245)	

Washing hands with soap after toilet	Yes	39 (29.8%)	92 (70.2%)	1	
	No	75 (58.1%)	54 (41.9%)	3.276 (1.963–5.470)	3.342 (1.939–5.761)

Trimming finger nails	Yes	39 (30.5%)	89 (69.5%)	1	
	No	75 (56.8%)	57 (43.2%)	3.003 (1.803–5.001)	2.884 (1.682–4.945)

Shoe wearing habit	Always	92 (42%)	127 (58%)	1	
	Sometimes	22 (53.7%)	19 (46.3%)	1.598 (0.818–3.123)	

Main source of water for washing clothes and cooking	Well	11 (57.9%)	8 (42.1%)	1.842 (0.715,4.744)	
	Pipe	103 (42.7%)	138 (57.3%)	1	

**Table 3:** Antimicrobial susceptibility pattern of *Salmonella* and *Shigella* isolate among street food handlers in Jimma town, southwest Ethiopia, Oct–Dec 2020.

Antibiotics	Susceptibility for *Salmonella*			Susceptibility for *Shigella*		
	S (%)	I (%)	R (%)	S (%)	I (%)	R (%)
Ampicillin (10 *μ*g)	0 (0)	0 (0)	21(100%)	0 (0)	0 (0)	2 (100%)
Ciprofloxacin (5 *μ*g)	20 (95.2%)	0 (0)	1(4.8%)	2 (100%)	0 (0)	0 (0)
Trimethoprim-sulphamethoxazole (23.75/1.25 *μ*g)	17 (81%)	0 (0)	3 (14.3%)	2 (100%)	0 (0)	(0)
Ceftriaxone(30 *μ*g)	21 (100%)	0 (0)	0 (0)	2 (2%)	0 (0)	0 (0)
Gentamicin (10 *μ*g)	19 (90.5%)	0 (0)	2 (9.5%)	2 (100%)	0 (0)	0 (0)
Doxycycline (30 *μ*g)	21 (100%)	0 (0)	0 (0)	2 (100%)	0 (0)	0 (0)

S=susceptible, I=intermediate resistance, and R = resistance.

## Data Availability

All the datasets on which the conclusions of the paper rely are presented in the main manuscript.
